# Retinal Thickness Deviation: A New OCT Parameter for Assessing Diabetic Macular Edema

**DOI:** 10.3390/jcm12123976

**Published:** 2023-06-11

**Authors:** Paola Marolo, Enrico Borrelli, Francesco Gelormini, Giacomo Boscia, Guglielmo Parisi, Matteo Fallico, Costanza Barresi, Giorgio Lari, Alessandro Berni, Francesco Bandello, Michele Reibaldi

**Affiliations:** 1 Department of Ophthalmology, University of Turin, 10126 Turin, Italy; francesco.gelormini@hotmail.it (F.G.); bosciagiacomo@gmail.com (G.B.); guglielmoparisi@gmail.com (G.P.); 2 Ophthalmology Department, Vita-Salute San Raffaele University, 20132 Milan, Italy; borrelli.enrico@yahoo.it (E.B.); barresi.costanza@hsr.it (C.B.); giorgiolari4@gmail.com (G.L.); berni.alessandro@hsr.it (A.B.); bandello.francesco@hsr.it (F.B.); 3 Department of Ophthalmology, University of Catania, 95123 Catania, Italy; matteofallico@hotmail.com

**Keywords:** diabetic macular edema, diabetes, visual acuity, optical coherence tomography, macular thickness, retinal thickness, anti-VEGF, corticosteroid, deviation

## Abstract

(1) Purpose: To determine the “retinal thickness deviation” (RTD) in diabetic macular edema (DME) eyes treated with intravitreal therapy and to establish associations between RTD and best-corrected visual acuity (BCVA). (2) Methods: We conducted a retrospective study, including consecutive patients with DME eyes undergoing intravitreal therapy with two years of follow-up. BCVA and central subfield thickness (CST) were collected at baseline and at 12 months and 24 months of follow-up. RTD was calculated as the absolute difference between measured and normative CST values at each time point. Linear regression analyses were performed between RTD and BCVA and between CST and BCVA. (3) Results: One hundred and four eyes were included in the analysis. The RTD was 177.0 (117.2) μm at baseline, 97.0 (99.7) μm at 12 months and 89.9 (75.3) μm at 24 months of follow-up (*p* < 0.001). RTD showed a moderate association with BCVA at baseline (R^2^ = 0.134, *p* < 0.001) and 12 months (R^2^ = 0.197, *p* < 0.001) and a substantial association at 24 months (R^2^ = 0.272, *p* < 0.001). The CST showed a moderate association with BCVA at baseline (R^2^ = 0.132, *p* < 0.001) and 12 months (R^2^ = 0.136, *p* < 0.001), while the association was weak at 24 months (R^2^ = 0.065, *p* = 0.009). (4) Conclusions: RTD showed a good association with visual outcome in patients with DME eyes undergoing intravitreal treatment.

## 1. Introduction

The efficacy of intravitreal anti-vascular endothelial growth factor (anti-VEGF) and corticosteroid therapies to treat diabetic macular edema (DME) relies on the measurement of best-corrected visual acuity (BCVA), which is considered the primary outcome in most clinical trials and in clinical settings [1,2,3,4].The assessment of retinal thickness change after treatment is based on structural optical coherence tomography (OCT) and, in particular, on the central subfield thickness (CST) measurement. In general, CST is the preferred analysis method to evaluate macular thickness change in trials involving DME patients [5].

However, the association between CST and BCVA proved to be moderate for both treatment-naïve and previously treated DME eyes [6,7]. A reduction in CST does not always correlate with an improvement in BCVA, especially when CST values fall below normal thresholds [2,6,7,8]. This means that a central retinal thinning below normal values could be associated with a poorer visual outcome. A post hoc analysis of data from the DRCR Retina Network Protocol T trial showed that CST and BCVA improvements after anti-VEGF therapy were not equal [7]. Accordingly, treatment with intravitreal corticosteroids with or without anti-VEGF therapy revealed that despite a reduction in retinal thickness the BCVA may be stable under a CST threshold [2,3]. Finally, long-term results of a 5-year DME treatment demonstrated a worsening of BCVA in cases with excessive retinal thinning [4].

On this basis, it appears that both an increase and a decrease in macular thickness in relation to normative values could be associated with worse values of visual acuity. Both a thicker-than-normal and a thinner-than-normal CST could be related to poorer visual outcomes. In this study, we introduced a novel parameter, which was named “retinal thickness deviation” (RTD), which indicates the deviation of CST from normative values. The purpose of this study was to determine the RTD in DME eyes that had been previously treated with intravitreal therapies and to evaluate its correlation with visual outcome.

## 2. Materials and Methods

### 2.1. Study Design

We conducted a retrospective cohort study on consecutive patients affected by DME who had a baseline assessment between December 2017 and December 2019. Included subjects were identified from the medical records of two different Medical Retina Units in Northern Italy: one based at “Città della Salute e della Scienza” Hospital, University of Turin, Turin and one at “Istituto di Ricovero e Cura a Carattere Scientifico (IRCCS) San Raffaele” Hospital, Vita-Salute San Raffaele University, Milan. The study protocol complied with the tenets of the Declaration of Helsinki and was reviewed and approved by the institutional ethics committees.

Inclusion criteria were as follows: age ≥ 18 years old; type 1 or type 2 diabetes; center-involved DME [9,10] previously treated with either intravitreal anti-VEGF or intravitreal dexamethasone implant; baseline Snellen equivalent BCVA ≤ 20/32 and ≥ 20/320 (≥0.20 and ≤1.20 logMAR); baseline CST ≥ 2 standard deviations thicker than sex-specific normal values (males ≥ 320 µm, females ≥ 305 µm on Heidelberg Spectralis) [1]; and follow-up of at least 24 months. In those cases in which either eye was eligible, one eye was randomly selected.

Exclusion criteria included the following: ocular conditions other than DME that may affect BCVA, including significant cataract graded more than N03 or NC3 according to the Lens Opacity Classification Scheme [11], other macular disorders, such as epiretinal membrane and foveal atrophy, and optic nerve diseases such as glaucoma; ocular conditions other than diabetic retinopathy occurring during the course of the study (vein occlusion, ocular inflammation, ocular hypertension and neovascular glaucoma); untreated proliferative diabetic retinopathy; refractive error (spherical equivalent) more than ±5 diopters or a history of amblyopia; and intravitreal anti-VEGF therapy in the 3 months before the baseline visit, intravitreal dexamethasone implant or laser treatment or any intraocular surgery in the 6 months before the baseline visit.

All patients received anti-VEGF intravitreal injections of bevacizumab, ranibizumab and aflibercept or an intravitreal implant of dexamethasone and were treated using either a pro re nata (PRN) or treat-and-extend regimen at the discretion of the treating physician.

The main objective of the study was to determine the RTD in DME eyes undergoing intravitreal therapy and to establish associations between RTD and BCVA, while the secondary objective was to determine associations between CST and BCVA in the same eyes.

### 2.2. Assessments

BCVA and CST were collected at baseline and at 12-month and 24-month follow-up visits. Patient charts were reviewed for demographic data, type of diabetes (type 1 or type 2), type of retinopathy (nonproliferative or proliferative), insulin use (yes or no), prior treatment for DME, including laser of intravitreal therapy (yes or no), lens status (phakic or pseudophakic), intraocular pressure and axial length. Visual acuities were tested using a LogMAR (logarithm of the minimal angle of resolution) chart.

### 2.3. OCT Grading

OCT imaging was performed using the Heidelberg Spectralis HRA + OCT (Heidelberg Engineering, Heidelberg, Germany) spectral domain (SD) OCT with a volumetric scan protocol composed of 19 horizontal B-scans, with 24× averaging for each B-scan, covering approximately a 5.5 × 4.5 mm area (20° × 15°) centered on the fovea. To be included, scans were also required to have a minimum signal strength index of 25, as advised by the manufacturer [12].

OCT images at the baseline study visit were first reviewed for eligibility by two independent readers (GB and FG). Subsequently, the two readers met to assess their agreement, and disagreements were resolved by further discussion and open adjudication to yield a single result for each case. For those cases in which the two readers were unable to reach an agreement on a single consensus result, the final decision was made by a third senior retinal expert (EB).

Images were also analyzed to generate quantitative measurements. Specifically, the retinal thickness was measured using the Spectralis instrument software within the Early Treatment Diabetic Retinopathy Study (ETDRS)-grid circle centered on the fovea [13]. Retinal thickness measurements were automatically averaged within the 1 mm diameter foveal subfield and inner ETDRS anulus located between 1 and 3 mm diameter circles. This algorithm automatically identifies the inner limiting membrane (ILM) and Bruch’s membrane and calculates the “retinal thickness” between these two boundaries. Before computing the thickness values, the readers evaluated all B-scans and manually corrected any segmentation or decentration errors.

### 2.4. Retinal Thickness Deviation

In order to quantify the deviation of retinal thicknesses from normative values, the RTD value was calculated as the absolute difference between measured and normative CST values as indicated in the following formula:

(retinal thickness deviation = |measured retinal thickness-normative retinal thickness|)(1)

The normative data were extracted from an age-adapted normative dataset obtained with the same device (i.e., Heidelberg Spectralis) and reported in a previous study [14].

### 2.5. Statistical Analysis

Statistical calculations were performed using Statistical Package for Social Sciences (version 20.0, SPSS Inc., Chicago, IL, USA). To detect departures from the normality distribution, a Shapiro–Wilk’s test was performed for all variables. A *p* value of 0.05 was considered statistically significant.

All quantitative variables are reported as mean and standard deviation (SD) in the Section 3 and Tables. BCVA, CST and RTD at the different times of follow-up (baseline and 12 and 24 months) were compared by analysis of variance. If significant, multiple comparisons were performed with the Tukey HSD test. To explore the association between OCT variables and visual acuity, we computed a linear regression analysis investigating the association between RTD and BCVA at baseline and at 12 and 24 months of follow-up. Subsequently, we investigated the same relationship between CST and BCVA at the same time points.

Positive (thickness above normal thickness values) and negative (thickness below normal thickness values) deviation subgroup analyses with linear regression were also performed to investigate the relationship between RTD and BCVA, as well as CST and BCVA, at 24 months.

For each association, the R^2^ coefficient was calculated in order to measure the “goodness of fit”, and it was interpreted according to Cohen [15] as follows:

- R^2^ < 0.02—very weak;
- 0.02 ≤ R^2^ < 0.13—weak;
- 0.13 ≤ R^2^ < 0.26—moderate;
- R^2^ ≥ 0.26—substantial.

## 3. Results

One hundred and four eyes of 104 patients (59 males (56.7%); mean age: 64.8 ± 14.8 years; range: 29 to 91 years) met the inclusion criteria and were included in the analysis. Table 1 summarizes the demographic and clinical characteristics of this study cohort.

The mean BCVA was 0.49 ± 0.23 LogMAR at baseline, 0.38 ± 0.24 LogMAR at 12 months and 0.33 ± 0.23 LogMAR at 24 months of follow-up. BCVA significantly improved after treatment (*p* < 0.001, ANOVA) at both 12-month (*p* < 0.001, Tukey HSD test) and 24-month follow-up visits (*p* = 0.002, Tukey HSD test). Similarly, a significant CST reduction was observed after treatment (*p* < 0.001, ANOVA). The mean CST was 442.6 ± 117.0 μm at baseline, 345.9 ± 113.5 μm at 12 months (*p* < 0.001, Tukey HSD test) and 238.7 ± 113.5 μm at 24 months (*p* < 0.001, Tukey HSD test) of follow-up.

### 3.1. Retinal Thickness Deviation

The mean RTD was 177.0 ± 117.2 μm, 97.0 ± 99.7 μm and 89.9 ± 75.3 μm at baseline, 12-month follow-up and 24-month follow-up, respectively. A significant reduction in RTD was found after treatment (*p* < 0.001, ANOVA) at both 12-month (*p* < 0.001, Tukey HSD test) and 24-month follow-up visits (*p* < 0.001, Tukey HSD test).

**Figure 1:**
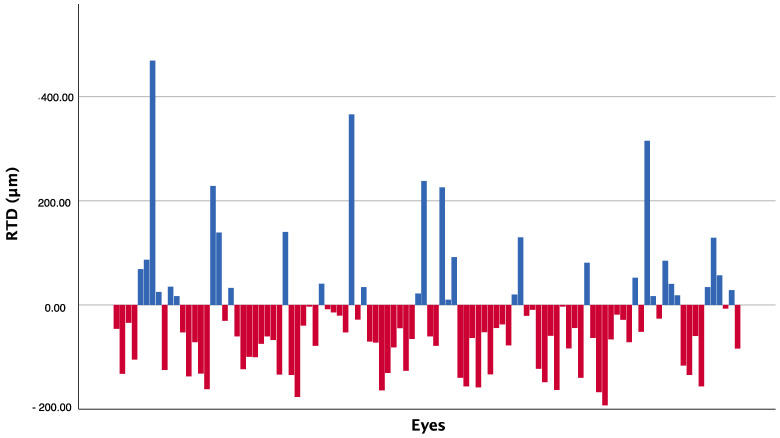
Retinal thickness deviation (RTD) for each of the 104 eyes at 24 months, differentiated as augmented (above the 0.00 RTD line, blue columns) or reduced (below the 0.00 RTD line, red columns) from normal retinal thickness.

At the 24-month follow-up visit, 73 out of 104 eyes (70.2%) showed a negative deviation from normal retinal thickness values, while 31 eyes (29.8%) showed a positive deviation. Figure 1 represents the deviation from normative retinal thickness data, differentiated by increased or reduced thickness, obtained for each single eye of the study at the last follow-up.

### 3.2. Retinal Thickness Deviation and Best-Corrected Visual Acuity

**Figure 2:**
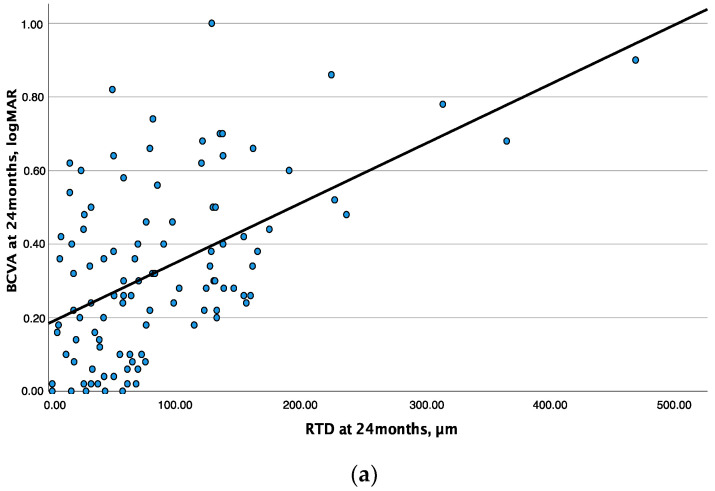

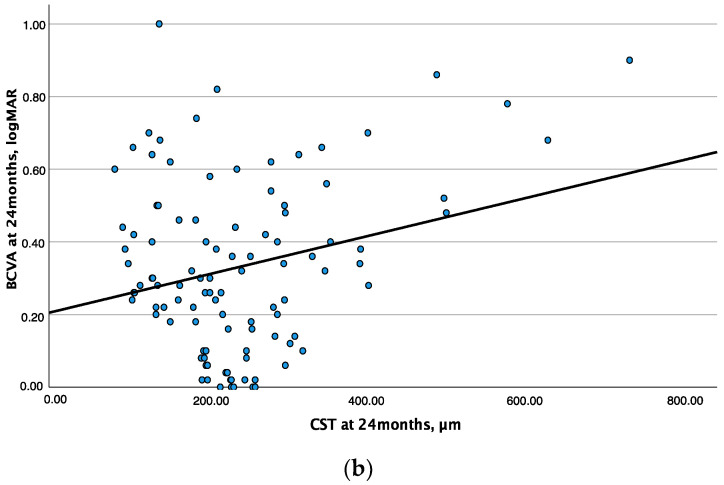
(**a**) Linear regression analysis between retinal thickness deviation (RTD) and best-corrected visual acuity (BCVA—set as dependent variable) at 24 months for a total of 104 eyes. The solid line indicates the line of best fit; R^2^ = 0.272. (**b**) Linear regression analysis between central subfield thickness (CST) and best-corrected visual acuity (BCVA—set as dependent variable) at 24 months for a total of 104 eyes. The solid line indicates the line of best fit; R^2^ = 0.065.

The linear regression analysis between RTD and BCVA showed a moderate association at baseline and the 12-month follow-up visit (R^2^ = 0.134, β = 0.001, *p* < 0.001 and R^2^ = 0.197, β = 0.001, *p* < 0.001, respectively) and a substantial association (R^2^ = 0.272, β = 0.002, *p* < 0.001) at the 24-month follow-up visit (Figure 2a).

### 3.3. Central Subfield Thickness and Best-Corrected Visual Acuity

The relationship between CST and BCVA obtained with the linear regression analysis showed a moderate association at both baseline and the 12-month follow-up visit (R^2^ = 0.132, β = 0.001, *p* < 0.001 and R^2^ = 0.136, β = 0.001, *p* < 0.001, respectively), while this association was weak (R^2^ = 0.065, β = 0.001, *p* = 0.009) at the 24-month follow-up visit (Figure 2b).

### 3.4. Subgroup Analysis

**Figure 3:**
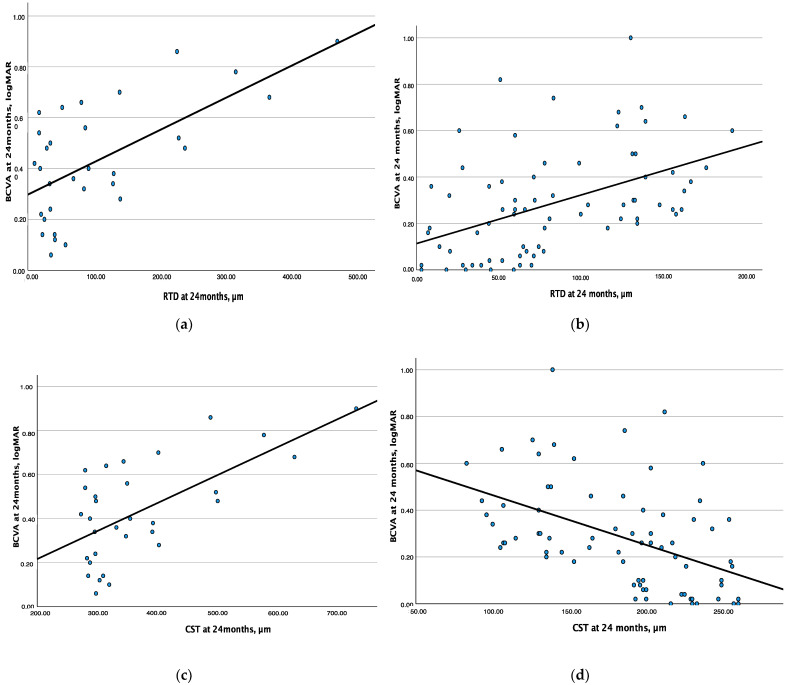
(**a**) Positive deviation subgroup (n = 31 eyes). Linear regression analysis between retinal thickness deviation (RTD) and best-corrected visual acuity (BCVA) at 24 months: direct linear correlation. (**b**) Negative deviation subgroup (n = 73 eyes). Linear regression analysis between retinal thickness deviation (RTD) and best-corrected visual acuity (BCVA) at 24 months: direct linear correlation. (**c**) Positive deviation subgroup (n = 31 eyes). Linear regression analysis between central subfield thickness (CST) and best-corrected visual acuity (BCVA) at 24 months: direct linear correlation. (**d**) Negative deviation subgroup (n = 73 eyes). Linear regression analysis between central subfield thickness (CST) and best-corrected visual acuity (BCVA) at 24 months: inverse linear correlation.

Taking into consideration the results described above, we also conducted a sub-analysis after dividing our study cohort into two subgroups according to the presence of the positive (thickness above normative thickness values) vs. negative deviation (thickness below normative thickness values) of CST values at the 24-month follow-up visit. Linear regression analyses between RTD and BCVA, as well as between CST and BCVA, were performed for each group of eyes. A total of 31 eyes and 73 eyes were included in the subgroup with positive RTD and in the subgroup with negative RDT, respectively. RTD and BCVA were characterized by a direct linear association in either subgroup of eyes (substantial association in eyes with positive deviation: R^2^ = 0.406, β = 0.001, *p* < 0.001; moderate association in eyes with negative deviation: R^2^ = 0.218, β = 0.002, *p* < 0.001) (Figure 3a,b). On the contrary, CST and BCVA showed a direct linear association in the subgroup of eyes with a positive deviation (substantial association: R^2^ = 0.405, β = 0.001, *p* < 0.001), while these two parameters were characterized by a negative linear association in the subgroup of eyes with a negative deviation (moderate association: R^2^ = 0.223, β = −0.002, *p* < 0.001) (Figure 3c,d).

## 4. Discussion

In this study, we investigated DME eyes undergoing intravitreal therapy over a period of 2 years looking at a novel parameter to evaluate retinal thickness change, namely, retinal thickness deviation. Our findings showed a substantial association between RTD and visual outcome at 24 months, while CST was weakly associated with visual outcome at the same time point.

Central subfield thickness is widely used in clinical trials and clinical practice to assess the efficacy of intravitreal therapy in patients with DME [1,2,4,5]. However, previous reports demonstrated that CST and BCVA may fail to show a linear association in DME eyes [6,7]. In particular, a reduction in CST below normative healthy values does not regularly result in a BCVA improvement [6]. In agreement with these previous reports, our results showed a moderate association between CST and BCVA at baseline and the 12-month follow-up, while this association was weak at the 24-month follow-up. These findings seem to corroborate the concept that an initial decrease in retinal thickness secondary to fluid absorption is associated with visual improvement, while a further retinal thinning secondary to retinal neurodegeneration is associated with visual worsening.

Bonnin et al. [16] investigated the relationship between inner retinal thickness and visual function in DME eyes treated with anti-VEGF and suggested that a further thinning of inner retinal layers after DME resolution may be related to a poor visual outcome. Assuming that the survival of ganglion cells is dependent on VEGF [17], a progressive decrease in its levels could be associated with neuronal loss and a consequent reduction in inner retinal thickness [18]. It is also reported that the visual outcome in DME eyes is primarily related to functional loss due to neuronal damage and not only to the anatomical retinal change secondary to vasculopathy [19]. Therefore, the finding of a poorer visual outcome with reduced retinal thickness in diabetic eyes is not surprising. Alternatively, since photoreceptor damage may result in a chronically reduced input to the inner retina, the inner retinal thinning may be secondary to disorganized synaptic architecture and transneuronal degeneration over time [20]. In another report using structural OCT, Borrelli et al. [10] demonstrated that the presence of specific quantitative modifications to the outer retina was correlated with worse long-term visual acuity in DME eyes undergoing anti-VEGF treatment. In detail, the latter study enrolled patients with DME resolution after an extended follow-up (i.e., >5 years) after anti-VEGF therapy initiation. This study demonstrated that thicknesses of the foveal and parafoveal outer retina were significantly lower in subjects with worse long-term visual outcomes. Taken together, these findings may explain our results of a weak linear association between CST and visual outcomes in DME eyes at the 24-month follow-up.

Since changes in retinal thickness from normal values (either an increase or decrease) seem to correlate with worse visual function, we proposed a novel parameter reflecting the absolute deviation from normative values. Our aim was to understand whether RTD might adequately correlate with visual outcomes, providing a new parameter that could help to objectively assess the efficacy of treatment.

An increase in retinal thickness due to fluid accumulation or a decrease in retinal thickness secondary to neurodegeneration would yield a similar effect on the retinal thickness deviation variable, which simply reflects the difference from the thickness of a normal, healthy retina. To obtain this novel parameter (i.e., RTD), we adopted a reference normative dataset for macular thickness differentiated by age that was reported in a previous study [14]. Notably, we did find that RTD and BCVA are characterized by a moderate linear relationship at baseline and at the 12-month follow-up, while a substantial relationship between these two parameters was found at 24 months. These findings seem to suggest that a deviation in retinal thickness from normative values, regardless of whether this deviation is positive or negative, may affect visual outcome in DME patients treated with intravitreal therapy. In particular, this association is stronger at long-term follow-up, when greater retinal thinning is more likely to be secondary to a neurodegenerative process.

Looking at the results of the subgroup analysis, we can derive a possible clarification of why RTD better correlates with BCVA than CST at a longer time point. Specifically, in the negative deviation subgroup, a positive correlation between RTD and BCVA was shown, while the correlation between CST and BCVA was negative. This aspect can be motivated by the functional loss caused by neuronal damage that occurs when retinal thickness values are below normality. Visual function is impaired both in the presence of diabetic macular edema with increased retinal thickness and in the presence of diabetic neuronal damage and retinal atrophy with decreased retinal thickness. It is noteworthy that the deviation from normality appeared to have changes throughout the analyzed visits. In detail, values were positive in all our patients at baseline, while a greater percentage of patients had negative values at the 24-month (70.2%) follow-up visit. The presence of a great number of patients with CST below normal values may account for the presence of a weak direct association between CST and BCVA at the 24-month follow-up visit. In other words, while an initial thinning of the retina (during the first year of treatment) may be associated with an improvement in visual acuity, a subsequent further decrease in retinal thickness may be associated with a worsening of vision. Therefore, a variable that better reflects the difference from normal, the retinal thickness deviation, could provide a better reflection of, and perhaps linear relationship with, visual acuity.

The main limitation of our study is its retrospective nature. A prospective longitudinal evaluation of the retinal thickness in center-involved DME eyes should help shed further light on the relation between RTD and visual acuity in these eyes. Additionally, the normative database adopted takes into account age but not race. Because CST and BCVA are only reflections of central foveal status and function, further studies are needed to evaluate whether other functional and anatomical parameters such as macular volume or peripheral macular thickness [20] can give a better reflection of the entire visual function of the eye. Our study also has strengths, including the use of a single OCT device and scanning protocol and standardized visits over the 2 years of follow-up.

In summary, this study demonstrated that a novel OCT parameter, which is named “retinal thickness deviation”, is substantially associated with visual outcomes in DME eyes treated with intravitreal therapy on a medium- to long-term follow-up (i.e., 24 months). This novel parameter could help to objectively assess the efficacy of intravitreal therapy in DME eyes and could be of great value for both clinical and research purposes.

## Figures and Tables

**Table 1:** Characteristics at baseline for DME eyes included in the study.

Characteristic	Eyes Included, No. (%)
No eyes	104
Women	45 (43.3)
Age, mean (±SD)	64.8 (±14.8)
Type of diabetes	
1	18 (17.3)
2	86 (82.7)
Insulin used	
No	33 (31.7)
Yes	71 (68.3)
Prior treatment for DME (laser or intravitreal therapy)	40 (38.5)
Lens status	
Pseudophakic	34 (32.7)
IOP (mmHg), mean (±SD)	12.3 (±4.2)
BCVA (logMAR), mean (±SD)	0.49 (±0.2)
CST (µm), mean (±SD)	442.6 (±117.0)
RTD (µm), mean (±SD)	177.0 (±117.2)

SD: Standard deviation; IOP: intraocular pressure; BCVA: best-corrected visual acuity (logMAR (logarithm of the minimum angle of resolution)); CST: central subfield thickness; RTD: retinal thickness deviation.

## Data Availability

The data that support the findings of this study are available from the corresponding author upon reasonable request.
